# Deep Learning for the Detection and Classification of Diabetic Retinopathy with an Improved Activation Function

**DOI:** 10.3390/healthcare11010097

**Published:** 2022-12-28

**Authors:** Usharani Bhimavarapu, Gopi Battineni

**Affiliations:** 1 Department of Computer Science and Engineering, Koneru Lakshmaiah Education Foundation, Vaddeswaramm 522302, Andhra Pradesh, India; 2 Medical Informatics Centre, School of Medicinal and Health Products Sciences, University of Camerino, 62032 Camerino, Italy

**Keywords:** diabetic retinopathy, fundus images, CNNs, activation functions

## Abstract

Diabetic retinopathy (DR) is an eye disease triggered due to diabetes, which may lead to blindness. To prevent diabetic patients from becoming blind, early diagnosis and accurate detection of DR are vital. Deep learning models, such as convolutional neural networks (CNNs), are largely used in DR detection through the classification of blood vessel pixels from the remaining pixels. In this paper, an improved activation function was proposed for diagnosing DR from fundus images that automatically reduces loss and processing time. The DIARETDB0, DRIVE, CHASE, and Kaggle datasets were used to train and test the enhanced activation function in the different CNN models. The ResNet-152 model has the highest accuracy of 99.41% with the Kaggle dataset. This enhanced activation function is suitable for DR diagnosis from retinal fundus images.

## 1. Introduction

Blood sugar levels that are abnormal in the human body accumulate in blood vessels as glucose is converted into energy. Diabetic retinopathy (DR) develops when a patient has had diabetes for more than ten years. DR occurs due to high blood pressure and causes damage to the retina, and it damages the retinal vascularization, which may cause blindness and death. Ophthalmologists can only observe retinal vascular swelling by conducting fundoscopy tests, but these are time-consuming and expensive. By 2030, there are estimated to be 552 million diabetic patients worldwide, and DR is a leading cause of blindness [1,2].

Detecting and treating visual loss early is the key to preventing visual loss [3]. In severe cases, the vessels swell, leak fluid, or block blood vessels, which results in abnormal blood vessel growth and complete blindness. Microaneurysms, hemorrhages, and exudates are the main symptoms of DR on the retina. A lesion’s shape, size, and overall appearance determine its severity. Fundus photography is an ophthalmologic screening method for DR [4]. Preventing diabetes-related blindness is clinically effective and cost-effective with an automated assessment technique [5].

Ophthalmologists diagnose the presence and the severity of the DR through a visual assessment by direct examination and evaluation of the eyes. For large numbers of diabetic patients globally, this process is expensive and time-consuming [6]. DR severity and early diagnosis of the disease remain a challenge, with statistics among trained ophthalmologists varying substantially [7]. Moreover, 75% of DR patients live in underdeveloped regions where sufficient ophthalmologists and the infrastructure for detection are unavailable [8]. Global screening activities have been created to counter the proliferation of preventable eye diseases, but DR exists at too large a scale to detect and treat DR efficiently on an individual basis.

DR occurs due to high blood pressure and causes retina damage. It damages retinal vascularization, which may cause blindness and even death. Ophthalmologists can only observe retinal vascular swelling by conducting fundoscopy tests, but these are time-consuming and expensive. There is a need to automatically identify DR by examining retinal fundus images. It is reported that deep learning models are a practical approach for DR detection, which can better identify DR compared to ophthalmologists [9].

The convolutional neural network (CNN) is one of the main models of deep learning used to detect, predict, and classify medical images. This study aims to automatically detect DR by implementing the updated activation function for the CNN model. The proposed new activation function is compared with other activation functions on the publicly available datasets DIARETDB0, DRIVE, CHASE, and Kaggle. The current CNN version has been improved by adding a unique activation function, which provides excellent results.

Our contribution is to identify DR by examining retinal fundus images efficiently and accurately. In addition, the enhanced CNN model will be evaluated and demonstrated for its performance. The proposed model does not require any specialized, inaccessible, or costly equipment to grade the fundus images; it can be run on a PC or laptop with average processors. In addition to detection and classification, the proposed model accurately visualizes abnormal regions in the fundus images enabling a clinical review and verification of the automated diagnosis. Microaneurysm detection is difficult for ophthalmologists because of its small appearance.

## 2. Research Background

Millions of individuals worldwide experience vision impairment without proper predictive diagnosis and eye care. To address the shortfalls of the current diagnostic task, an automated solution for retinal disease diagnosis from fundus images is proposed [10]. This technique could alleviate the workloads of trained ophthalmologists, allowing untrained technicians to screen and process DR patients without dependence on clinicians.

Some studies adopted the CNN model with dropout regularization, augmentation, and preprocessing with different datasets and achieved a 94% accuracy [11]. In another study, the CNN model classifies the five-stage DR on the publicly available dataset and achieved high specificity and low sensitivity [12]. Three networks categorize the DR images as normal or abnormal and referable or nonreferrable DR. The first network implements the Inception model; the second recognizes the lesions, and the third network crops the DR images [13].

The CNN models, such as Inception V3, Dense 121, Xception, Dense 169, and ResNet 50, can automatically diagnose DR and corresponding phases [14,15]. In [16], the authors highlighted that the VGGNet model has the highest accuracy in DR classification. By adopting the EYEPACS dataset, three additional deep-learning models successfully classified DR [17]. In addition, the other CNN models, namely AlexNet and VGGNet-16, achieved an 83.68% accuracy with DR stage classification that was not explicitly classified [18].

The activation functions in the neural network activate the neurons of the neural network, and these mathematical functions, which are attached to the neurons, decide whether to fire the current neuron. The activation function introduces nonlinearity into the output neurons. A model without the activation function behaves like a linear regression. The activation function transforms the nonlinear input and makes it capable to learn and perform complex datasets with high accuracy. There are many existing activation functions in neural networks, which are further explained in Table 1.

Based on the different activation functions mentioned in the above table, we aim to implement the new activation function for CNN. A performance comparison was carried out with the achieved performance of the proposed new activation function with the other activation functions in the publicly available dataset using DIARETDB0. The goal is to provide a highly effective, low-cost solution to DR detection without depending on clinicians to examine and grade images manually.

A fully automated CNN model could process thousands of heterogeneous fundus images accurately for DR detection. In other words, it eliminates the need for resource-intensive manual fundus image analysis across clinical settings and guides high-risk patients to further care. We present an improved activation function-based CNN model applied to the publicly available diabetic retinopathy datasets DIARETDB0, DRIVE, CHASE, and Kaggle diabetic retinopathy.

## 3. Materials and Methods

### 3.1. Dataset

**Figure 1:**
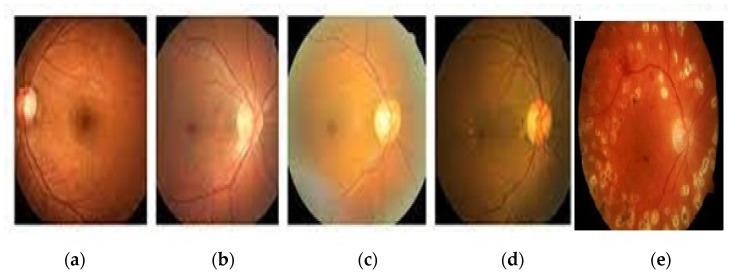
(**a**) Class 0 (No DR), (**b**) Class 1 (mild nonproliferative retinopathy), (**c**) Class 2 (moderate nonproliferative retinopathy), (**d**) Class 3 (severe nonproliferative retinopathy), and (**e**) Class 4 (proliferative DR).

In this study, we used the datasets DIARETDB0, DRIVE, CHASE, and Kaggle. There are 130 images in the DIARETDB0 [19] dataset, 110 of which are used for training, while 20 images are used for testing. In DRIVE [20], we selected 34 images for training and eight for testing from a set of 40 color fundus images. This included 33 images without DR and seven with early DR signs. In CHASE [21], 28 retinal fundus images were used for training, and four were used for testing. There are 88,702 images in the Kaggle [22] dataset. We used 75,397 images for training and 13,305 images for testing. We found 25,810 with no DR, 2443 with mild DR, 5292 with moderate DR, 873 with severe DR, and 708 with proliferative DIARETDB0 consisting of five different classes that are visualized in Figure 1.

**Figure 2:**
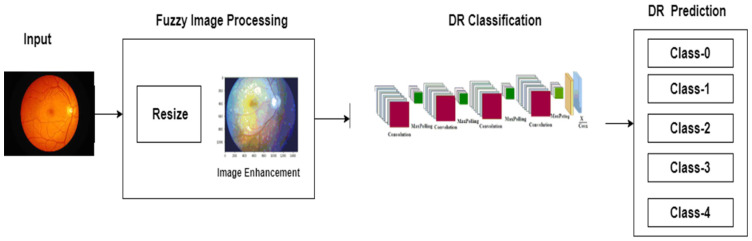
Experimental framework.

DR fundus images will be classified into various severity levels with high accuracy in the present study. DR severity can be assessed using an automated model, and the modified CNN architecture increases the accuracy of categorizing diabetic retinopathy. The experimental framework can be observed in Figure 2.

### 3.2. Image Preprocessing

Four datasets, namely DIARETDB0, DRIVE, CHASE, and Kaggle, were considered to classify DR images. The preprocessing phase removes imperfections from retinal images, improves image quality, and allows spatial domain techniques to operate on pixels. In addition to their efficiency in computation, spatial domain techniques require less processing power. The pixel values were directly used as input information in pixel-based approaches. This enhancement technique relies on the grey levels to enhance the high-contrast image produced by the pixel-based approach. To effectively process the image in the next stage, spatial domain techniques were used in the preprocessing phase [23]. To improve image quality, fuzzy set type II was applied in the preprocessing step, and the image was fuzzified by the equation given as

(1) $$\mu \prime \left(g_{\mathrm{ij}}\right)=\frac{g-g_{m}}{g_{\max}-g_{\min}}$$

The upper and the lower ranges of type II fuzzy membership functions were assessed as follows:

The upper membership functions

(2) $$\mu^{\mathrm{upper}}={\left[\mu \left(x\right)\right]}^{\alpha }$$

The lower membership functions

(3) $$\mu^{\mathrm{lower}}={\left[\mu \left(x\right)\right]}^{1/\alpha },\;\propto =0.9,\;0<\alpha \leq 1$$

where ∝ is the image color level ranges from 0 to max-1. g_max_ and g_min_ are the maximum and minimum image color levels. The contrast-enhanced image depends on the value of ∝; when ∝ increases, then, the image contrast also increases.

If ∝ = 0.9, the lesions are brighter, and the enhanced image has a shadier background. It is possible to achieve these goals with higher values and membership values, and the enhanced image is improved as a result. To find the membership values, Hamacher T co-norm was applied (Equation (4))

(4) $$\mu^{\mathrm{enhanced}}\left(g_{\mathrm{ij}}\right)=\frac{\mu^{\mathrm{upper}}+\mu^{\mathrm{lower}}+\left(\lambda -2\right)\mu^{\mathrm{upper}}.{\;\mu }^{\mathrm{lower}}}{1-\left(1-\lambda \right)\mu^{\mathrm{Upper}}.{\;\mu }^{\mathrm{lower}}}$$

where λ = average of the image.

### 3.3. Improved CNN Model Training

**Figure 3:**
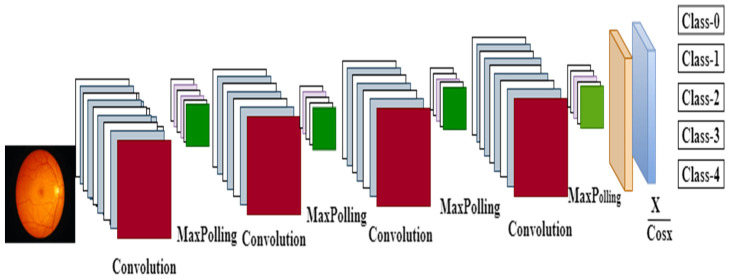
CNN with improved activation function.

To improve the contrast of the retina fundus image, we resized it to 32 × 32 pixels to reduce the complexity of the image. Following feature extraction, the CNN will be trained until convergence, and then the DR classification will be tested to determine its accuracy. Based on lesion detection and segmentation, convolution layers extracted features for correlated tasks and improved DR classification performance [24,25]. Figure 3 shows the improved CNN model architecture.

When training the DR fundus image, it is necessary to adjust the hyperparameters to enhance performance. Layer one of the DR learns the edges of the fundus image, while layer two learns the classification of the fundus image. Using the updated, improved activation function, the max pooling layer reduces overfitting with a kernel size of 3 × 3 and a stride of 1 × 1 on dense layers. By applying the convolution layer to the different spatial positions, each convolution layer generates a single-feature map using backpropagation during training.

By using the average coefficient in the subsampling layer, we trained the bias and weight. Since the CNN has so many free parameters, and because distortion has invariant characteristics, it is suitable for DR classification because of its low computational time during the training phase. For testing, we applied four convolutions and four pooling layers and two fully connected layers with improved activation functions. Several filters with specific coefficient values were employed in every convolution layer, and maximum pooling was used in the pooling layer. By default, the CNN extracts implicit and invariant features of distortion, so the CNN is suitable for DR classification.

#### 3.3.1. Convolution Layer

The fundus image matrix and filter are inputs to the convolution layer. Receptive fields and shared weights are used by CNNs to recognize images. By extracting parts of the fundus image and invoking receptive fields, a convolution layer detects it. Although CNN feature maps share the same weights and biases, the way they are generated differs from application to application, and these shared values represent the same features in fundus images. To extract the features of the fundus images, the activation map was used.

#### 3.3.2. Pooling Layer

A max-pooling layer was applied, which is a nonlinear down-sampling technique that divides the activation map in half and collects the maximum value in each half. This layer removes information in the appropriate areas of the image based on the generated features found in the image. The pooling layer reduces parameters and computation in the network to prevent overfitting.

#### 3.3.3. Activation Function

The proposed improved activation function has more sparsity in the hidden units; by this feature, the CNN can be trained efficiently and compared to the Sigmoid and the remaining activation functions. During the testing phase, we observed more of a loss reduction and a lower processing time than the standard activation functions. The proposed activation and its first derivative are presented in Equation (5).

(5) $$\frac{d}{\mathrm{dx}}\left(\frac{x}{\cos x}\right)=\frac{\cos x+\mathrm{xsinx}}{\cos^{2}x}$$

#### 3.3.4. Fully Connected Layer

A fully connected layer exists after all the convolution and the pooling layers. This layer takes all the neurons from the last pooling layer and converts them into a one-dimensional layer. After multiple layers, the final layer is the proposed activation function, followed by the fully connected layer. The properties of the proposed activation function are listed as follows:

- f(0) = 0 and $f^{\prime }$(0) = 1
  f(x) is derivable ∀x∈R
     Proof: f (0^−^) = f (0^+^) = 0
     $f^{\prime }$ (0^−^) = $f^{\prime }$ (0^+^) = 1
     f(x) is derivable ∀x∈R
- When x > 0, f(x) > 0 and $f^{\prime }$(x) = 1
     Proof: $\forall x\in R,\frac{x}{\cos x}\in$[−1,1]
     when x > 0, f(x) > 0 and $f^{\prime }$(x) = 1
     f(x) = $\frac{x}{\cos x}$
     *=* $\frac{\cos x+x\sin x}{\cos^{2}x}$
     0 < f(x) < x and $f^{\prime }$(x) > 0
- As x → +∞, f(x) → 0
     Proof: As x → +∞, f(x) → 0
     As x → +∞, f(x) → 1

The proposed improved activation function has more sparsity in the hidden units, and by this feature, the CNN can be trained efficiently compared to the Sigmoid and the remaining activation functions. The improved activation function avoids the saturation conditions and normalizes the input during the training. During the testing phase, the loss and the processing time were reduced more than the standard activation functions. The improved activation function avoids the saturation conditions, and the gradient does not become zero and normalizes the input during the training.

## 4. Results and Discussion

### 4.1. Accuracy Comparison of Different Activation Functions

We tested using various activation functions, such as ReLu, SoftMax, Swish, and Mish, on the diabetic retinopathy DiaretDB0 dataset with epochs 5000, learning rate = 0.01 and batch size 64, and Nadam Optimiser. Experimentation was conducted on different hidden layers with the proposed activation function, Nadam optimizer, and dense layers, with a batch size of 64. As shown in Table 2, Table 3 and Table 4, we tested the different activation functions with the proposed one in terms of epochs, learning rates, and batch sizes. We implemented the updated activation function using a Keras backend. With the suggested activation function, experiments were conducted on different epoch numbers. With high epochs, the proposed activation function provided the highest accuracy. Even for many epochs, the suggested activation function performs well.

With a fixed learning rate, the performance of the proposed model was tabulated in terms of accuracy. With a learning rate of le-2, Tanh yields a value of 91%. As a result of setting the learning rate to l × 10^−2^, the ReLU value is 93%. When l × 10^−3^ is set for ELU, a 95% accuracy is achieved. SELU recorded 97% for a learning rate of l × 10^−3^, while Sigmoid recorded 91%.

**Figure 4:**
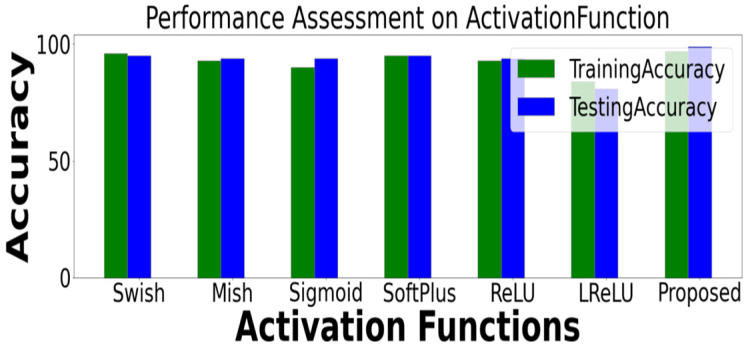
Accuracy comparison of different activation functions related to the proposed one.

The activation function used in this study had an epoch size of 5000, a learning rate of 1 × 10^−2^, and batch sizes of 8, 16, 32, 64, 128, 256, 512, 1024, and 2048 (Table 4). Multiple experiments were conducted with different hyperparameters on the dataset during the training process. The accuracy comparison of different activation functions is displayed in Figure 4. In the diabetic retinopathy model, the proposed activation function gives the most accurate results for the dense layers. Compared to ReLu, LReLu, Sigmoid, and Softplus, Mish and Swish’s activation functions provide a near-consistent improvement.

### 4.2. CNN Model Performance Evaluations

As mentioned, five-class DR images based on grading were fed to CNN models including Inception-v3, VGG-19, ResNet-50, AlexNet, GoogleNet, SqueezeNet, and ResNet-152. The performance of the enhanced CNN with the activation function was compared to other adopted models. Table 5 presents the assessment of the distinct model performance metrics on four adopted datasets. The proposed model outperforms the others in terms of testing accuracy. The number of layers in VGG-19 is 19; ResNet-50 is 50 layers; SqueezeNet is 18; GoogleNet is 22; AlexNet is 8, and Inception V3 is 48. For the benchmark datasets DiaretDB0, DRIVE, CHASE, and Kaggle, our proposed model had the lowest model loss. Based on the results, the enhanced CNN can detect and classify DR with an appropriate testing loss.

Five-class DR images based on grading were fed to CNN models including Inception-v3, VGG-19, ResNet-50, AlexNet, GoogleNet, SqueezeNet, and ResNet-152 using the existing activation functions SELU, ReLu, Sigmoid, and ELU. The performance of the existing activation function over different topologies was compared and tabulated in Table 5. The proposed model outperforms the others in terms of testing accuracy, model loss, and processing time for the Kaggle dataset. Based on the results, the proposed activation function can detect with a low loss and less processing time. Table 6 tabulates the comparison of the loss and the processing time for the proposed activation function with the different CNN models using the different activation functions.

**Figure 5:**
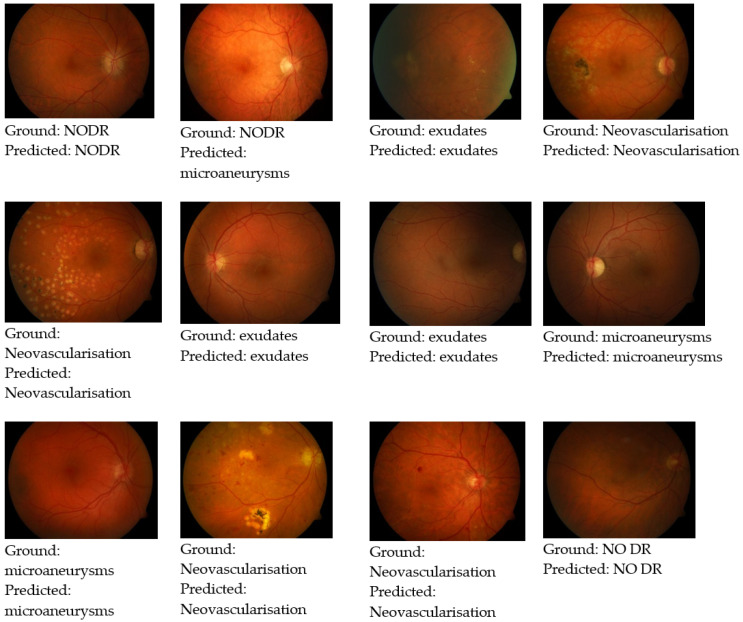
Test images with ground truth with improved CNN predictions.

Based on the enhanced CNN, the prediction output reflects the probability and accuracy of the correct predictions. In Figure 5, the ground-truth images are shown along with enhanced CNN predictions.

Different activation functions were tested on the DR datasets with epochs 5000, learning rate = 1 × 10^−2^ and batch size 64, and Nadam Optimizer. The proposed activation function outperforms existing activation functions with an accuracy of 96.64%, a sensitivity of 97.96%, and a specificity of 98.79% on the DIARETDB0 dataset. In terms of loss, the proposed activation function achieved a reduced loss of 0.0010. Table 4 tabulates the loss values from the experiments using the proposed activation function with the various pre-trained networks on the DIARETDB0, DRIVE, CHASE, and Kaggle datasets. From the experimental results, the ResNet-152 network performs better, achieving 0.0013 in the DIARETDB0 dataset, 0.0015in DRIVE, 0.0017in CHASE, and 0.0010 in the Kaggle dataset.

We compared our proposed model with some existing methodologies on the DiaretDB0 dataset. The proposed activation function achieved the highest 0.93 AUC score compared with existing works [26,27]. On the DRIVE dataset, it achieved a 0.94 AUC score, which is better than the functions described in [28,29]. Additionally, the CHASE dataset had a higher AUC score of 0.97 than [30,31]. The Kaggle dataset achieved a maximum AUC of 0.99, which is more than previous studies [32,33]. On the Kaggle dataset, the proposed activation function achieved the highest accuracy of 99.41%. According to the experimental results, the proposed model has a better accuracy of 99.41%, a sensitivity of 98.28%, and a specificity of 99.94% of the Kaggle dataset. This is followed by AlexNet with an accuracy of 96.27, a sensitivity of 87.64, and a specificity of 96.89, while SqueezeNet has the least accuracy of 87.85.

## 5. Conclusions

We evaluated the performance of the enhanced CNN model using the DIARETDB0, DRIVE, CHASE, and Kaggle datasets. The image processing-based enhancement was performed using the improved CNN model. The DiarteDB0 dataset resulted in 96.6% classification accuracy; 97.96% sensitivity, 99.5% precision, and 99.1% F1 score; the DRIVE dataset resulted in 97.84% classification accuracy; 98.45% sensitivity, 99.68% precision, and 99.57% F1 score; the CHASE dataset, resulted in 99.05% classification accuracy, 98.45% sensitivity, 99.94% precision, and 99.89% F1 score, and the Kaggle dataset resulted in 99.41% classification accuracy; 98.28% sensitivity, 99.89% precision, and 99.93% F1 score. Using retina images, the proposed model efficiently diagnoses diabetic retinopathy. Comparing the proposed activation with traditional deep learning models, we found that it improved diagnosis and classification performance. Compared to previous classification techniques, the proposed improved activation function in the CNN model improves the accuracy and processing time. Due to the enhanced activation function utilized in the enhanced CNN model, the model’s processing time is reduced by approximately 7 ms by avoiding the inseparable classification of nonlinear data. As compared to existing methods, the proposed activation function enhanced the classification of diabetic retinopathy by 99.41%.

## Figures and Tables

**Table 1:** Different activation functions and definitions.

Function	Definition	Equation	Limitations
Linear type	The final activation function of the last layer is just a linear function of the first layer of the input, and it can be used in the output layer.	Y = x; −∞ to +∞	Nonlinearity is difficult to achieve.
Binary type	The binary classification is used mainly when inputs exceed thresholds, otherwise, outputs are zero.	0; if input < threshold, otherwise 1; if input > threshold; Range: {0, 1}	Cannot classify the multiclass problems
Nonlinear			
Sigmoid	A small change in input will result in a large change in output. To convert the output into a predictable score, this layer is placed at the end of the model.	1/(1 + e^x^); Range:0 to1 or −1 to 1	During training, a model other than the output layer is invalid due to the vanishing gradients
Tanh	It is used as an alternative to the Sigmoid function if the output is other than zero and one.	Tanh(x = (e^x^ − e^−x^)/(e^x^ + e^−x^); Range: −1 to +1	If the weighted sum of the input is very large, then the function gradient becomes very small and close to zero. It has the vanishing gradient problem.
ReLu	It is implemented in the hidden layers of the model. It is computationally less expensive and much faster than the tanh and Sigmoid and solves the vanishing gradient problem	max (0, x); if x is positive, output x, otherwise 0; Range: 0 to +∞	It does not compute the exponentials and the divisions. It overfits more than the Sigmoid function. It does not avoid the exploding gradient problem.
Swish	It deals with the vanishing gradient problem. It helps in normalizing the output. The output does not saturate to a maximum value, i.e., the gradient does not become zero.	x.σ(x); Range: −∞ to +∞	It is computationally more expensive than the Sigmoid.
Mish	It is continuously differentiable and nonmonotonic. It is used in the hidden layer.	x.tanh(ln(1 + e^x^)); Range: −∞ to +∞	It is computationally more expensive than the ReLu.

**Table 2:** Comparison of accuracy proposed with state-of-the-art activation functions on different epochs.

Activation Function	Epochs								
	100	200	300	400	500	600	700	800	900
Tanh	0.95	0.96	0.96	0.96	0.96	0.96	0.97	0.97	0.97
Sigmoid	0.95	0.95	0.95	0.95	0.95	0.95	0.95	0.95	0.95
Relu	0.95	0.96	0.96	0.96	0.96	0.96	0.96	0.96	0.96
LReLu	0.95	0.95	0.95	0.95	0.96	0.96	0.96	0.96	0.96
ELU	0.95	0.96	0.96	0.96	0.96	0.96	0.96	0.96	0.96
SELU	0.98	0.98	0.99	0.99	0.99	0.99	0.99	0.99	0.99
Log sin	0.95	0.95	0.95	0.95	0.95	0.95	0.95	0.95	0.95
Sinc	0.96	0.96	0.96	0.96	0.96	0.96	0.97	0.97	0.97
Wave	0.94	0.94	0.94	0.94	0.95	0.95	0.95	0.95	0.95
Rootsig	0.96	0.96	0.96	0.96	0.97	0.97	0.97	0.97	0.97
Logsigm	0.96	0.96	0.96	0.96	0.96	0.96	0.96	0.96	0.96
Proposed	0.96	0.96	0.96	0.97	0.97	0.97	0.97	0.98	0.98

**Table 3:** Accuracy comparison of proposed function with others on different learning rates.

Activation Function	Learning Rates								
	1 × 10^−1^	1 × 10^−2^	1 × 10^−3^	1 × 10^−4^	1 × 10^−5^	1 × 10^−6^	1 × 10^−7^	1 × 10^−8^	1 × 10^−9^
Tanh	0.91	0.91	0.91	0.91	0.92	0.92	0.93	0.93	0.94
Sigmoid	0.95	0.95	0.95	0.95	0.95	0.95	0.94	0.94	0.94
Relu	0.93	0.93	0.93	0.94	0.94	0.95	0.95	0.95	0.94
LReLu	0.95	0.95	0.95	0.95	0.95	0.95	0.95	0.95	0.95
ELU	0.95	0.95	0.95	0.95	0.95	0.95	0.95	0.95	0.95
SELU	0.98	0.97	0.97	0.97	0.97	0.97	0.97	0.97	0.97
Log sin	0.95	0.95	0.95	0.95	0.95	0.95	0.95	0.95	0.94
Sinc	0.96	0.96	0.96	0.96	0.96	0.97	0.96	0.97	0.96
Wave	0.94	0.94	0.94	0.94	0.94	0.94	0.94	0.94	0.94
Rootsig	0.96	0.96	0.96	0.96	0.96	0.96	0.96	0.95	0.95
Logsigm	0.96	0.96	0.96	0.97	0.96	0.97	0.96	0.96	0.96
Proposed	0.98	**0.99**	0.98	0.98	0.98	0.98	0.97	0.97	0.97

**Table 4:** Comparison of accuracy proposed with state-of-the-art activation functions on different batch sizes.

Activation Function	Batch Sizes								
	8	16	32	64	128	256	512	1024	2048
Tanh	0.95	0.96	0.96	0.96	0.96	0.96	0.97	0.97	0.97
Sigmoid	0.95	0.95	0.95	0.95	0.95	0.95	0.95	0.95	0.95
Relu	0.95	0.96	0.96	0.96	0.96	0.96	0.96	0.96	0.96
LReLu	0.95	0.95	0.95	0.95	0.96	0.96	0.96	0.96	0.96
ELU	0.95	0.96	0.96	0.96	0.96	0.96	0.96	0.96	0.96
SELU	0.98	0.98	0.98	0.99	0.99	0.99	0.99	0.99	0.99
Log sin	0.95	0.95	0.95	0.95	0.95	0.95	0.95	0.95	0.95
Sinc	0.96	0.96	0.96	0.96	0.96	0.96	0.97	0.97	0.97
Wave	0.94	0.94	0.94	0.94	0.95	0.95	0.95	0.95	0.95
Rootsig	0.96	0.96	0.96	0.96	0.97	0.97	0.97	0.97	0.97
Logsigm	0.96	0.96	0.96	0.96	0.96	0.96	0.96	0.96	0.96
Proposed	0.98	0.98	0.98	0.99	0.98	0.98	0.97	0.97	0.97

**Table 5:** Performance comparison of different CNN models with proposed activation function on different datasets.

Database	Model	Accuracy	Sensitivity	Specificity	Precision	F1 Score	AUC	Model Loss
DIRATEDB0	Inception-v3	92.12	94.53	95.41	92.76	95.57	0.83	0.0029
	VGG-19	94.92	97.56	98.34	95.25	94.77	0.73	0.0025
	ResNet-50	93.54	95.27	98.32	99.43	98.42	0.89	0.0019
	AlexNet	95.82	81.62	94.36	91.66	94.47	0.79	0.0021
	GoogleNet	94.08	78.36	92.42	89.22	90.39	0.78	0.0029
	SqueezeNet	84.52	89.46	96.86	91.38	89.33	0.70	0.0058
	ResNet-152	96.64	97.96	98.79	99.53	99.15	0.93	0.0013
Kaggle	Inception-v3	93.63	96.34	96.74	93.63	94.52	0.89	0.0026
	VGG-19	93.32	97.24	93.77	96.74	96.62	0.95	0.0024
	ResNet-50	94.64	94.24	96.86	95.74	97.72	0.97	0.0016
	Alexnet	96.27	87.64	96.89	97.84	98.78	0.87	0.0020
	GoogleNet	95.87	83.33	93.85	94.79	94.83	0.88	0.0024
	SqueezeNet	87.85	90.36	97.36	93.92	91.88	0.84	0.0030
	ResNet-152	99.41	98.28	99.94	99.89	99.93	0.98	0.0010
DRIVE	Inception-v3	96.43	93.74	93.63	93.62	96.53	0.88	0.0036
	VGG-19	92.45	93.74	94.63	98.44	97.22	0.84	0.0047
	ResNet-50	92.44	93.72	95.27	94.83	95.88	0.93	0.0023
	AlexNet	96.74	86.89	95.84	93.83	97.62	0.74	0.0032
	GoogleNet	93.88	77.92	95.24	85.68	93.73	0.73	0.0034
	SqueezeNet	86.07	86.35	93.46	93.77	90.69	0.74	0.0046
	ResNet-152	97.84	98.45	99.26	99.68	99.57	0.94	0.0015
CHASE	Inception-v3	94.65	96.34	94.63	96.62	93.34	0.85	0.0025
	VGG-19	93.74	94.83	95.85	93.62	96.62	0.94	0.0027
	ResNet-50	93.83	93.22	96.95	95.73	94.68	0.96	0.0028
	AlexNet	96.62	88.74	97.83	94.38	92.67	0.84	0.0028
	GoogleNet	92. 58	79.48	97.28	90.82	93.73	0.84	0.0038
	SqueezeNet	88. 42	90.84	98.25	94.84	91.73	0.78	0.0047
	ResNet-152	99.05	98.45	99.59	99.94	99.89	0.97	0.0017

**Table 6:** Performance comparison of different existing activation functions with proposed activation function on Kaggle datasets.

Activation Function	Model	Accuracy	Processing Time	Model Loss
SELU	Inception-v3	91.82	20	0.0029
	VGG-19	91.18	22	0.0026
	ResNet-50	92.17	20	0.0020
	AlexNet	93.28	20	0.0021
	GoogleNet	92.27	19	0.0028
	SqueezeNet	84.94	22	0.0036
	ResNet-152	98.57	17	0.0015
ReLu	Inception-v3	90.82	21	0.0028
	VGG-19	90.83	24	0.0027
	ResNet-50	91.28	26	0.0026
	AlexNet	92.72	22	0.0021
	GoogleNet	91.26	21	0.0025
	SqueezeNet	82.17	23	0.0032
	ResNet-152	95.73	19	0.0020
Sigmoid	Inception-v3	90.63	22	0.0034
	VGG-19	90.37	25	0.0027
	ResNet-50	92.62	26	0.0021
	AlexNet	91.63	23	0.0026
	GoogleNet	90.68	22	0.0026
	SqueezeNet	82.73	23	0.0036
	ResNet-152	95.63	20	0.0016
ELU	Inception-v3	90.52	23	0.0029
	VGG-19	90.26	25	0.0028
	ResNet-50	92.47	27	0.0028
	AlexNet	92.95	21	0.0027
	GoogleNet	91.63	20	0.0026
	SqueezeNet	83.53	21	0.0034
	ResNet-152	96.63	19	0.0021
Proposed	Inception-v3	93.63	15	0.0026
	VGG-19	93.32	16	0.0024
	ResNet-50	94.64	14	0.0016
	AlexNet	96.27	16	0.0020
	GoogleNet	95.87	14	0.0024
	SqueezeNet	87.85	15	0.0030
	ResNet-152	99.41	07	0.0010

## Data Availability

Not applicable.
